# Pleasure Boatyard Soils are Often Highly Contaminated

**DOI:** 10.1007/s00267-014-0249-3

**Published:** 2014-02-23

**Authors:** Britta Eklund, David Eklund

**Affiliations:** Department of Applied Environmental Science (ITM), Stockholm University, 106 91 Stockholm, Sweden

**Keywords:** Antifouling paint, Boatyards, Metals, TBT (tributyltin), PCBs, PAHs

## Abstract

The contamination in pleasure boatyards has been investigated. Measured concentrations of copper, zinc, lead, mercury, cadmium, tributyltin (TBT), the 16 most common polycyclic aromatic hydrocarbons (∑16 PAHs), and the seven most common polychlorinated biphenyls (∑7 PCBs) from investigations at 34 boatyards along the Swedish coast have been compiled. The maximum concentrations were 7,700 for Cu, 10,200, for Zn, 40,100 for Pb, 188 for Hg, 18 for Cd, 107 for TBT, 630 for carcinogenic PAHs, 1,480 for ∑16 PAHs, and 3.8 mg/kg DW for ∑7 PCB; all 10–2,000 higher than the Swedish environmental qualitative guidelines. In addition, the mean of the median values found at the 34 places shows that the lower guidance value for sensitive use of land was exceeded for the ∑7 PCBs, carcinogenic PAHs, TBT, Pb, Hg, and Cu by a factor of 380, 6.8, 3.6, 2.9, 2.2 and 1.7, respectively. The even higher guideline value for industrial use was exceeded for the ∑7 PCBs and TBT by a factor of 15 and 1.8, respectively. TBT, PAHs, Pb, Cd, and Hg are prioritized substances in the European Water Framework Directive and should be phased out as quickly as possible. Because of the risk of leakage from boatyards, precautions should be taken. The high concentrations measured are considered to be dangerous for the environment and human health and highlight the urgent need for developing and enforcing pleasure boat maintenance guidelines to minimize further soil and nearby water contamination.

**Electronic supplementary material:**

The online version of this article (doi:10.1007/s00267-014-0249-3) contains supplementary material, which is available to authorized users.

## Introduction

A large number of people appreciate owning and using a pleasure boat. The International Council of Marine Industry Associations (ICOMIA) estimated that approximately 6 million boats exist in Europe (ICOMIA 2007). Due to the large archipelago along the Swedish coast, nearly 1 million boats are found only in Sweden (Swedish Transport Agency 2010). Thus, nearly 1 million boats are stored and maintained on land during the winter. Almost another two million boats exist in Finland, Denmark, and Norway meaning almost half of European leisure boats are found in the Nordic countries (Eklund et al 2013). Boat maintenance usually includes scraping and sandpapering the boat hull in the spring and painting before launching with an antifouling paint that contains toxicologically active substances. After the sailing season, the loose paint fragments are removed by washing and scraping before the hull is repainted the next spring. Boat motors also need maintenance, which can result in accidental fuel and oil spills. In addition, harmful substances may be released during other boat repair activities that are performed in boatyards.

As long as humans have been sailing, fouling by attaching organisms has been known as a problem that needs to be overcome. Much fouling organisms lower the speed through the water, and severe fouling may impair the maneuverability. Biofouling may increase the fuel consumption up to 40 % (Yebra et al. 2004). To protect the boat from fouling already the Phoenicians, 700 BC, started to use lead sheathing (Lunn 1974). Also, plates of copper (Cu)or zinc (Zn) have been used as sheathing of boat hulls. Eventually, the method of grinding the metals and instead making paint formulations became common in the 20th century. Besides lead (Pb), Zn, and Cu, arsenic, antimony, and mercury (Hg) were used in antifouling coatings (Lunn 1974). Due to its harmful environmental and human impacts, the use of lead has been restricted since 1976 (Directive 76/769/EEC, Directive 89/677/EEC, UN environment program 2008). Today, it is almost exclusively used for protecting boats of historical value. Restricted use of Hg was enforced in 1979 (79/117/EEG).

Among the metals, copper has been the most extensively used active agent in antifouling paints in the last century, which may contain copper between 4 and 30 % of the paint (Brooks and Waldock 2009; Thomas and Brooks 2010). In Sweden, different regulations for use of antifouling paints containing copper as active agent were enforced in 2001 (Swedish Chemical Agency 1998). No paints with copper as active agent were approved for use on the East coast of Sweden, whereas a certain leakage rate was allowed for use on the West coast. This regulation resulted in the development and marketing of a number of so-called physical working paints (eroding paints) for use on the East coast. However, studies on leakage from such paints revealed high leakage of Zn (Ytreberg et al 2010), which was shown to be toxic to Baltic Sea organisms (Karlsson and Eklund 2004, Karlsson et al 2006, 2010).

Experiments with toxic organic compounds in antifouling paints resulted in a formulation with tributyltin (TBT) as active agent. Because of its high efficiency for preventing the attachment of fouling organisms to boat hulls, TBT became the most commonly used active ingredient in antifouling paints in the 1970s and 80s (Fent 2006; Antizar-Ladislao 2008). TBT was correlated to endocrine disturbances in mollusks (for example, Alzieu et al. 1986, 1989; Bryan et al. 1986, 1987; Gibbs et al. 1986, 1988), and this discovery led to restrictions in EU, USA, Canada, Australia, and New Zealand (Antizar-Ladislao 2008 and references therein; Dafforn et al 2011). In addition, triphenyltin (TPhT) causing imposex similar to TBT is also commonly used in some countries, for example, Japan (Horiguchi et al. 1997). EU banned the use of organic tin compounds as active agents in paints for use on boats shorter than 25 m in 1989 (Directive 89/677/EEC). The International Maritime Organization (IMO) agreed to prohibit the use of organotin compounds in antifouling paints on all ships in the International Convention on the control of harmful antifouling systems on ships (AFS convention IMO 2001). This agreement was ratified by the European Union in 2003 (Directive 2002/62/EG, EC/782 2003). The AFS convention criteria were fulfilled in September 2007 and were fully enforced in September 2008. Europe took an even more aggressive step and banned any TBT-painted ships from European harbors as of January 1, 2008 (EC/782 2003). This action shows that society reacted on the serious negative effects of this substance. In addition, TBT is a priority hazardous substance under the Water Frame Directive (2000/60/EC) and should be eliminated as soon as possible (Directive 2008/105/EC). For a more in-depth review and history of the development of antifouling paints, see Thomas (2001) and Yebra et al. (2004).

Other sources of toxic substances that may be important in boatyards include oils and exhausts from fuel combustion [for example, polychlorinated biphenyls (PCBs) and polycyclic aromatic hydrocarbons (PAHs)].

The harmful impacts of the active ingredients used in antifouling paints have been extensively studied for the last few decades (e.g., Férnandez-Alba et al. 2002, Karlsson et al 2004, 2006). Recent reviews have been published by Fent (2006), Antizar-Ladislao (2008), and Thomas and Brooks (2010) regarding the leakage of toxic substance from boat hulls into water and its effect on non-target organisms. Turner and colleagues focused on paint particles and the distribution and transport of antifouling particles to the marine environment (Singh and Turner 2009a, b; Turner et al. 2008a, b) and their effects on different organisms (Turner et al. 2009a, b). In addition, Turner (2010) reviewed the marine pollution from antifouling paint particles. In our study, compiling the concentrations of antifouling paint pollution found in boatyards complements current knowledge regarding the extent of problematic residues and the distribution of harmful substances in paint particles.

The aims of this study were to compile data from investigations on the concentrations of dangerous substances found in pleasure boatyards, to compare the data with existing guidelines, and to estimate the extent of the problem.

## Materials and Methods

### Collection of Data

In this study, measured concentrations of metals and organic pollutants in soil at 34 boatyards along the Swedish coast have been compiled. The investigations were done by contacting the 66 municipalities located along the southern Swedish coast from the Norwegian border to 30 km north of Stockholm. A total of 34 boatyards in 21 municipalities had been investigated by 18 consulting companies. Since the purpose of the investigations differed, the design of the investigations and the number of analyzed samples varied. The sampling had in most cases been done by digging with a shovel; sometimes, a tractor had been used. Most commonly, the soil consisted of sand and gravel in the top 20–50 cm, with larger stones or clay deeper down. No fractionation of the samples had been done other than the removal of larger stones and particles in accordance with the procedure in the standards for analyses. In all cases, the samples had been sent for chemical analysis to accredited laboratories that used the standardized methods required by the Swedish EPA. All of the boatyards were investigated between 2000 and 2011 except for five in the City of Stockholm, which had been investigated in 1997. For a detailed description of individual reports, the interested is welcome to contact the municipality of the site of the respective investigation. (See supplementary material for contact information).

The reason for performing these 34 investigations was most often (18 sites) due to a planned change in land use (for example, building residential housing instead of using the area as a boatyard). However, in some cases, the local environmental authority wanted to classify the land risk, which required additional knowledge regarding the contamination situation.

No complete information exists on how long the places have been used as boatyards and neither any information exists on former use of the land.

### Selection and Structure of Data

The samples were taken at different depths and intervals in the 34 investigations. Since the surface sample was between 0 and 0.05 m in depth at some sites and between 0 and 1 m in depth at other sites, it was not possible to compare data from specific depths. The compounds analyzed varied in the investigations. Only the metals found most frequently were compiled in this paper, in other words, Cu, Zn, Pb, Hg, and Cd. Among the organic compounds are TBT, TPhT, carcinogenic polycyclic aromatic hydrocarbons (PAH canc.), and total polycyclic aromatic hydrocarbons (PAH tot) and PCBs also being compiled, where such data existed. The mean values with standard deviation are presented along with the number of samples of each contaminant at each boatyard. The measured compound concentrations potentially vary by several orders of magnitude between different samples within each boatyard. Thus, we chose to also present the median data values from each site. In addition, the maximum values are presented to illustrate the levels that might be reached at boatyards and the potential risk of contaminating metals and organic substances.

### Guideline Values Used

For comparison, the existing recommended Swedish guideline values (SEPA www.naturvardsverket.se) were used. There are two guideline value levels: one for industrial use that is called “Less Sensitive Land” (LSL) and one for sensitive land use that is called “Sensitive Land” (SL). The SL level is intended for places where people live or spend time daily and where children play. In contrast, the LSL level is intended for land that is used for industrial purposes where higher concentrations of harmful substances are acceptable. The used threshold values for these levels are shown in Table 1.

**Table 1:** Summary of median, mean, and maximum concentrations of dangerous metals and organic substances measured in soil samples from 34 investigated boatyards in coastal communities in Sweden

Element/compund	Guideline values for SL (mg/kg DW)	Guideline values for LSL (mg/kg DW)	Median of all median concentrations	Mean of all median concentrations (mg/kg DW)		Mean of all mean concentrations (mg/kg DW)		Mean of all maximum concentrations (mg/kg DW)		Maximum concentrations measured in the entire dataset from 34 boatyards
				Mean	SD	Mean	SD	Mean	SD	(mg/kg DW)
Cu	80	200	52	*130*	210	**540**	1,100	**1,800**	2,200	**7,700**
Zn	250	500	112	180	170	*400*	390	**1,700**	2,000	**10,200**
Pb	50	400	35	*150*	270	**440**	940	**2,700**	6,800	**40,100**
Hg	0.25	2.5	0.05	*0.56*	1.7	**2.50**	7.30	**13**	35	**190**
Cd	0.5	15	0.19	0.35	0.37	*0.52*	0.53	*1.7*	3.2	**18**
TBT^a^	1	2	0.23	**3.7**	10	**7.0**	13	**16**	28	**110**
PAH canc.	0.3	7	1.30	*2.0*	2.0	**9.2**	18	**62**	150	**630**
∑16 PAH	7	45	2.29	5.0	9.0	*25*	35	**158**	343	**1,480**
∑7 PCB	0.008	0.2	0.30	**3.0**	5.7	**0.46**	5.6	**6.0**	8.2	**27**

For comparison, the Swedish guideline values for Sensitive Land use (SL) and Less Sensitive Land use (LSL) are shown. Values higher than SL are marked in italics and higher than LSL are marked in bold

^a^No guideline value for TBT in soil exists in Sweden. The values in the table are based on recommendation in Finland (Finnish directive 2007)

In Sweden, there is no guidance for TBT in soil. Thus, in this compilation, comparison has been made with guidance values in Finland (Finnish directive 2007). The Finnish values include both TBT and triphenyltin (TPhT) and are 1 and 2 mg kg DW for high and very high levels, respectively. Since TPhT is often very low relative to TBT and it was not always measured, we used the Finnish TBT values 1 and 2 mg kg DW as a substitute for SL and LSL.

Different data presentation methods have been used for PAHs in the various original reports. Today, the PAHs are defined by their molecular weight as low (PAH L), medium (PAH M), or high (PAH H). However, when the investigations were conducted, it was common practice to present the data as a sum of the 16 most commonly analyzed PAHs (∑16 PAHs) with the carcinogenic fraction of the PAHs. Since most of the investigations used this method for presenting data, we adopted this method for our comparisons. Based on the Swedish recommendations, the environmental SL and LSL guidelines for carcinogenic PAHs are 0.3 and 7 mg/kg DW, respectively. Similarly, the environmental SL and LSL guidelines for ∑16 PAHs are 7 and 45 mg/kg DW, respectively. The carcinogenic compounds analyzed in this study include benz[*a*]anthracene, chrysene, benzo[*b*]fluoranthene, benzo[*k*]fluoranthene, benzo[*a*]pyrene, dibenz(a,h)anthracene, and indeno(1,2,3-cd)pyrene. For the 16 compounds, naphthalene, acenaphthene, acenaphthylene, fluorene, phenanthrene, anthracene, fluoranthene, benzo[*ghi*]perylene, and pyrene were also included.

The Swedish guideline values that were used for PCBs were 0.008 mg/kg DW for SL and 0.2 mg/kg DW for LSL. These values include the sum of the seven PCB congeners (28, 52, 101, 118, 138, 153, and 180), such as ∑7 PCB.

For depth information for a particular sample, we refer to the original report, which may be achieved by contacting the respective municipality (see supplementary material for contact information).

## Results

When appropriate, the concentrations above the LSL guideline concentrations are denoted with figures in bold in the tables and the concentrations that are between the SL and the LSL concentrations are denoted with figures in italic.

### Summary for the Entire Dataset

The mean values of median, mean, and maximum results of the entire dataset are summarized in Table 1, and the potential risk of contaminants at boatyards is illustrated by the maximum values found for each element/compound. The maximum sample concentrations are several orders of magnitude higher than both the SL and LSL concentrations for all substances. In one boatyard, the maximum measured concentration of ∑7 PCB was 27 mg/kg DW. Thus, the guideline for SL and LSL concentration is exceeded by a factor of 3375 and 135, respectively. The corresponding factors by which the maximum measured sample concentrations exceed the SL and LSL threshold concentrations were 2,100 and 90 for carcinogenic PAHs, 800 and 100 for Pb, 760 and 76 for Hg, 211 and 33 for ∑16 PAHs, 107 and 54 for TBT, 40 and 20 for Zn, and 36 and 1.2 for Cd.

The mean of the maximum concentration for all data from each site showed that very high levels of contaminants could be found at boatyards and all contaminants were higher than both SL and LSL.

The mean of all mean values from the boatyards investigated showed higher values than LSL by all contaminants except for Cd which only was higher than SL (Table 1).

The mean of the median values from each site shows that TBT and ∑7 PCB exceeded LSL but Cu, Pb, Hg, and carcinogenic PAHs only exceeded SL, and the Zn, Cd, and ∑16 PAH values were lower than the guideline values (Table 1).

Finally, if the median values of all median values are compared to the guideline values none of the contaminants are higher than these (Table 1).

### Number of Samples from the Investigated Boatyards

Most of the sites were sampled for metals (Table 2). The number of samples varied between 1 and 65, and the mean number of samples from each site was 11, 12, 12, 10, and 11 for Cu, Zn, Pb, Hg, and Cd, respectively. The organic substances were sampled less frequently (Table 3). ∑16 PAHs were sampled at 26 sites and carcinogenic PAHs at 22 sites. The mean number of samples was 11 and 8, respectively, and the range was 1–50 samples per boatyard. TBT and TPhT were investigated at only 17 and 13 sites, respectively, in other words, about half the sites, with a mean of 7 (TBT) and 8 (TPhT) samples per site and a variation between 1 and 30 samples. ∑7 PCB was sampled even less and data are available from only ten of the sites, with a mean of seven samples per site and 18 samples at one site, the highest number.

**Table 2:** Metals

Boatyard no	Year of investigation	Depth of samples (m)	Copper (Cu)			Zinc (Zn)			Lead (Pb)			Mercury (Hg)			Cadmium Cd(VI)		
			*N*	Mean (mg/kg DW)	SD	N	Mean (mg/kg DW)	SD	*N*	Mean (mg/kg DW)	SD	*N*	Mean (mg/kg DW)	SD	*N*	Mean (mg/kg DW)	SD
1	2008	0–1	5	*149*	6	5	196	138	5	35	7.3	5	–		5	0.16	0.06
2	2007, 2009	0–1.25	10	**1,178**	1,253	10	**808**	704	10	*390*	420	10	–		10	*1.00*	0.93
3	2003	0–0.05	4	**1,133**	2,047	4	**831**	1,317	4	*56*	76	4	0.08	0.04	4	0.21	0.16
4	2003	0–0.5	5	**502**	641	5	*471*	489	5	*350*	461	5	3**.89**	6.48	5	0.45	0.34
5	2003	0–0.05	1	14		1	18		1	4		1	<0.001		1	<0.05	
6	2010	0-0.4	4	27	27	4	74	37	4	17	6	4	0.05	0.03	4	<0.2	
7	2008	0–1.2	12	**1,276**	2,378	12	**1,746**	3,263	12	**566**	1,376	12	**3.06**	7.24	12	*2.30*	5.21
8	2010	0–0.3	1	**240**		1	110		1	*91*		1	0.16		1	<0.19	
9	2008	0–2	7	21	8	7	43	17	7	13	8	7	–		7	<0.21	
10	2003	0–0.9	6	**311**	730	6	*455*	954	6	**526**	1,261	6	0.03	0.04	6	0.15	0.11
11	2010	0–0.5	3	49	13	3	*497*	232	3	*72*	42	3	*0.47*	0.30	3	*0.67*	0.09
12	2010	0–2	55	*107*	138	65	212	261	65	*76*	148	58	*1.23*	2.49	48	0.17	0.22
13	2009	0–0.15	2	**6,400**	1,838		–	–		**975**	177		–			–	
14	2011	0–1	27	18	40	60	92	90	60	*68*	148	27	*1.10*	3.38	27	0.16	0.13
15	2010	0–1	0	–		0	–		0	–		0	–		0	–	
16	2009	0–3.75	19	*107*	232	19	122	228	19	*150*	253	19	*1.43*	2.40	19	0.45	0.34
17	2007	0–2	11	*80*	73	11	*490*	652	11	*122*	132	11	0.04	0.05	11	*0.54*	0.32
18	2006	0–0.05	5	34	39	5	116	104	5	*348*	42	5	*0.25*	0.47	5	<0.2	
19	2005	0–0.55	12	**738**	1,288	12	**1,378**	1,838	12	**5,396**	11,829	12	–		12	*1.88*	1.69
20	2009	0–1.1	9	*92*	95	9	120	99	9	*89*	128	9	*1.54*	4.34	9	0.28	0.57
21	2002, 2004, 2006	0–0.5	17	**649**	1,490	17	**520**	936	17	**425**	1,248	11	*1.22*	1.73	17	0.27	0.43
22	2007	0–1.5	12	**559**	605	12	*351*	345	12	*215*	311	12	*1.03*	2.30	12	0.25	0.23
23	2003	0–0.7	5	*162*	182	5	82	41	5	*109*	144	5	*0.49*	1.00	5	0.14	0.08
24	2004, 11	0**–**2	28	**258**	976	28	*315*	558	28	*222*	562	11	*0.63*	1.58	28	0.22	0.45
25	2011	0–0.3	21	62	213	21	*257*	273	21	*212*	465	10	0.16	0.01	21	*1.30*	1.19
26	2006	0–0.2	14	**312**	660	14	226	413	14	**491**	1,323	14	**4.17**	8.23	14	0.34	0.39
27	2008	0-0.6	17	**276**	330	17	*388*	845	17	*309*	663	17	*1.87*	4.82	17	0.43	0.67
28	1997	0–3.5	10	**1,627**	1,707	10	**953**	1,057	10	**1,602**	1,924	10	**39.92**	62.88	10	*0.75*	0.41
29	1997	0–0.7	10	**698**	1,049	10	*468*	439	10	**495**	541	10	**3.67**	4.72	10	*0.76*	0.57
30	1997	0–2.5	10	**303**	249	10	*402*	281	10	**1,023**	1,384	10	**2.95**	3.36	10	<1	
31	1997	0–2.5	9	49	18	9	180	200	9	34	13	9	0.09	0.08	9	*0.83*	1.00
32	1997	0–2.1	10	*163*	207	10	**588**	967	10	*296*	522	10	*0.70*	0.68	10	*1.08*	0.99
33	2006	0–1.0	2	13	13	2	43	61	2	16	20	2	0.03	0.04	2	0.09	0.01
34	2008	0–2	14	60	127	14	*273*	290	14	*60*	104	2	0.24	0.49	14	0.35	0.37

The mean and standard deviations of selected metals in soil at 34 investigated boatyards in Swedish coastal municipalities. Values higher than the Swedish guideline values for sensitive land use (SL) are marked in italics and higher than less sensitive land use (LSL) are marked in bold. *N* denotes the number of samples. – data missing

Boatyards; *1* Hålkedalskilen, Strömstad; *2* Nilssons båtbyggeri, Strömstad; *3* Råssö, Strömstad; *4* Källviken, Uddevalla; *5* Dragsmark östra, Uddevalla; *6* Orust marina, Orust; *7* Rönnäng 1:75, Tjörn; *8* Rönnäng 1:153, Tjörn; *9* Toftenäs, Tjörn; *10* Vattenfall, Stenungsund; *11* Röd 1:73, Öckerö, *12* Fiskebäcks hamn, Göteborg; *13* Skintebo bryggor, Göteborg; *14* Bryggan 1, Halmstad; *15* Lomma hamn, Lomma; *16* Dragör, Malmö; *17* Skillinge hamn, Simrishamn; *18* Angelskog, Ronneby; *19* Hästö, Norrköping; *20* Lindö, Norrköping; *21* Strandängen, Nyköping; *22* Femöre 1:4, Oxelösund; *23* Bo Klok, Nynäshamn; *24* Tyresö strand 1:36, Tyresö; *25* Fisksätra marina, Nacka; *26* Kilsviken, Nacka; *27* Skutviken, Nacka; *28* Vikingarnas Segelsällskap, Stockholm; *29* Margretelund; Stockholm; *30* Årstaviken, Stockholm; *31* Fiskarfjärden, Skärholmen, Stockholm; *32* Göta Segelsällskap, Stockholm; *33* Björkdungens förskola, Danderyd; *34* Cirkusplatsen, Östhammar. For the exact depth for each sample, we refer to the original reports or the corresponding author

**Table 3:** Organic compounds

Boatyard no	Year of investigation	Depth of samples (m)	TBT			TPhT			PAH canc.			∑16 PAH			∑7 PCB		
			*N*	Mean (mg/kg DW)	SD	*N*	Mean (mg/kg DW)	SD	*N*	Mean (mg/kg DW)	SD	*N*	Mean (mg/kg DW)	SD	*N*	Mean (mg/kg DW)	SD
1	2008	0–2.4	12	**3.52**	11.00	12	0.13	0.290	2	*1.28*	1.13	2	1.93		0		
2	2007, 09	0–0.5	8	**7.64**	13.30	8	0.14	0.18	2	*0.97*	0.53	2	1.90		0		
3	2003	0–0.05	0						2	0.03	0.02	2	0.03	0.04	2	<0.03	
4	2003	0–0.5	0						2	**11.04**	10.40	2	*21.20*	6.48	2	**17.32**	9.48
5	2003	0–0.05	0						0			0			0		
6	2010	0–0.4	1	0.28	0.00	1	<0.001		0			4	0.62	0.03	2	<0.008	
7	2008	0–0.6	6	**35.85**	48.47	6	0.03	0.03	7	**35.92**	61.83	7	**67.58**	7.24	5	**0.76**	0.61
8	2010	0–0.3	0						2	<1.1		2	0.68		0		
9	2008	0–2	4	0.01	0.02				7	*1.12*	1.43	7	2.18		0		
10	2003	0–0.9	0						0			0		0.04	0		
11	2010	0-0.5	3	<0.001		3	<0.001		0			3	**93.35**	92.66	0		
12	2010	0–2	10	0.53	0.82				32	*1.37*	2.83	32	2.56	2.49	18	**0.28**	0.45
13	2009	0–0.15	2	**40.10**	10.90	2	3.57	0.32	0			0			0		
14	2011	0–1	0						0			43	*31.04*	3.38	0		
15	2010	0–2	30	**3.10**	5.60	30	0.24	0.40	0			0			0		
16	2009	0–3.75	4	*1.98*	1.68				0			50	**58.78**	2.40	0		
17	2007	0–1	4	*1.50*	2.43	4	0.00	0.01	4	<0.0001		6	1.98	0.05	0		
18	2006	0–0.05	2	**5.67**	5.63	2	0.02	0.02	2	**10.30**	2.70	2	*28.30*	0.47	0		
19	2005	0–0.55	0						12	**73.85**	180	12	**156.55**	410	0		
20	2009	0–1.1	1	**2.89**		1	0.15	0.15	6	<0.002		6	1.97	4.34	7	*0.05*	0.09
21	2002, 2004, 2006	0–1	9	*1.04*	1.55				17	**15.12**	23.18	17	*30.95*	1.73	0		
22	2007	0–1.5	12	0.69	0.51	11	0.09	0.22	0			0		2.30	0		
23	2003	0–0.7	0						5	*1.02*	1.37	5	1.72	1.00	0		
24	2004, 2011	0–2	15	0.03	0.07	15	0.00	0.01	1	*1.40*		12	0.89	1.58	0		
25	2011	0–0.3	4	0.33	0.26	4	0.09	0.14	9	0.18	0.08	7	0.38	0.01	5	**0.33**	0.64
26	2006	0–0.2	0			0			10	*2.39*	2.94	10	3.98	8.23	0		
27	2008	0–0.6	0			0			16	*4.42*	4.08	0		4.82	16	**2.00**	1.35
28	1997	0-3.5	0			0			3	*1,13*	0.29	3	3.20	0.70	0		
29	1997	0–0.7	0			0			3	**12,50**	16.12	3	*40.67*	52.78	0		
30	1997	0–2.5	0			0			3	*1,43*	0.82	3	4.07	2.30	0		
31	1997	0–2.5	0			0			3	0,21	0.12	3	1.03	0.21	0		
32	1997	0–2.1	0			0			3	*3,82*	3.31	3	*11.33*	9.86	0		
33	2006	0–1.0	0			0			0			0		0.04	0		
34	2008	0–2	0			0			0			11	*15.77*	36.93	3	<0.001	

The mean and standard deviations of selected organic compounds in soil at 34 investigated boatyards in Swedish coastal municipalities. Values higher than the Swedish guideline values for sensitive land use (SL) are marked in italics and higher than less sensitive land use (LSL) are marked in bold. *N* denotes the number of samples. – data missing

Boatyards; *1* Hålkedalskilen, Strömstad; *2* Nilssons båtbyggeri, Strömstad; *3* Råssö, Strömstad; *4* Källviken, Uddevalla; *5* Dragsmark östra, Uddevalla; *6* Orust marina, Orust; *7* Rönnäng 1:75, Tjörn; *8* Rönnäng 1:153, Tjörn; *9* Toftenäs, Tjörn; *10* Vattenfall, Stenungsund; *11* Röd 1:73, Öckerö, *12* Fiskebäcks hamn, Göteborg; *13* Skintebo bryggor, Göteborg; *14* Bryggan 1, Halmstad; *15* Lomma hamn, Lomma; *16* Dragör, Malmö; *17* Skillinge hamn, Simrishamn; *18* Angelskog, Ronneby; *19* Hästö, Norrköping; *20* Lindö, Norrköping; *21* Strandängen, Nyköping; *22* Femöre 1:4, Oxelösund; *23* Bo Klok, Nynäshamn; *24* Tyresö strand 1:36, Tyresö; *25* Fisksätra marina, Nacka; *26* Kilsviken, Nacka; *27* Skutviken, Nacka; *28* Vikingarnas Segelsällskap, Stockholm; *29* Margretelund; Stockholm; *30* Årstaviken, Stockholm; *31* Fiskarfjärden, Skärholmen, Stockholm; *32* Göta Segelsällskap, Stockholm; *33* Björkdungens förskola, Danderyd; *34* Cirkusplatsen, Östhammar. For the exact depth for each sample, we refer to the original reports or the corresponding author

**Table 4:** The median and maximum concentrations of selected metals in soil at 34 investigated boatyards in Swedish coastal municipalities

Boatyard no	Cu Median (mg/kg DW)	Cu Max (mg/kg DW)	Zn Median (mg/kg DW)	Zn Max (mg/kg DW)	Pb Median (mg/kg DW)	Pb Max (mg/kg DW)	Hg Median (mg/kg DW)	Hg Max (mg/kg DW)	Cd(VI) Median (mg/kg DW)	Cd(VI) Max (mg/kg DW)
1	*130*	**260**	200	*410*	39	39	–	–	0.19	0.23
2	**750**	**3,500**	**725**	**2,000**	*245*	**1,000**	–	–	*0.80*	*2.40*
3	*150*	**4,200**	220	**2,800**	20	*170*	0.07	0.12	0.26	0.40
4	**220**	**1,500**	210	**1,100**	22	**890**	0.08	**15.00**	0.33	*0.90*
5	14	14	18	18	4	4	<0.001	<0.001	<0.05	<0.05
6	20	63	89	100	18	24	*0.05*	0.08	<0.20	<0.20
7	*103*	**8,100**	71	**10,200**	17	**4,800**	0.03	**25.00**	0.10	**18.00**
8	**240**	**240**	110	110	*91*	*91*	0.16	0.16	<0.19	<0.19
9	18	34	43	75	9	26	–	–	<0.21	<0.21
10	13	**1,800**	79	**2,400**	18	**3,100**	0.02	0.10	0.14	0.33
11	54	58	**560**	**690**	*53*	*120*	*0.52*	*0.74*	*0.67*	*0.75*
12	52	**560**	110	**1,270**	27	**955**	<0.1	**9.79**	<0.1	*0.82*
13	**6,400**	**7,700**	–	–	**975**	**1,100**	–	–	–	–
14	9	**214**	61	*429*	28	**840**	*0.45*	**18.00**	0.10	*0.50*
15	–	–	–	–	–	–	–	–	–	*–*
16	19	**790**	42	**1,000**	*52*	**950**	0.12	**8.40**	0.39	*1.40*
17	51	**200**	215	**2,300**	*58*	**420**	<0.04	0.17	*0.55*	*1.20*
18	26	*100*	83	*290*	18	*110*	<0.05	*1.10*	<0.2	<0.2
19	**285**	**4,600**	*305*	**5,000**	**530**	**40,100**	–	–	*1.60*	*4.40*
20	39	**268**	97	150	32	*381*	<1	**13.10**	0.08	0.29
21	25	**5,110**	89	**3,600**	27	**5,110**	*0.50*	**4.40**	0.10	*0.68*
22	**290**	**1,900**	199	**1,090**	*117*	**1,140**	0.05	**7.96**	0.16	*0.77*
23	69	**367**	86	125	37	*357*	<0.04	*2.27*	0.11	0.27
24	37	**5,190**	114	**2,630**	41	**2,880**	<1	**5.27**	<0.1	*2.00*
25	62	**660**	120	**1,000**	30	**2,000**	0.19	0.22	*0.86*	*3.10*
26	39	**2,283**	68	**1,593**	*50*	**5,001**	<1	**27.00**	<0.1	*0.60*
27	72	**1,000**	120	**3,600**	34	**2,700**	0.16	**20.00**	<0.1	*2.90*
28	**1,021**	**4,230**	**657**	**3,150**	**1,052**	**5,220**	**8.83**	**188.00**	<1	*1.49*
29	*127*	**3,120**	*260*	**1,380**	*344*	**1,940**	*2.14*	**14.90**	<1	*2.14*
30	**215**	**655**	*380*	**1,040**	**670**	**4,650**	*1.55*	**10.90**	<1	<1
31	48	*83*	119	**709**	35	*54*	<0.02	*0.25*	<1	*3.49*
32	75	**693**	*251*	**3,270**	*108*	**1,740**	*0.38*	*2.25*	<1	*3.59*
33	13	*22*	43	86	16	30	0.03	0.06	<0.05	0.13
34	15	**488**	109	**782**	17	*71*	<1	*1.64*	<0.1	*1.18*

Values higher than the Swedish guideline values for sensitive land use (SL) are marked in italics and higher than less sensitive land use (LSL) are marked in bold. The median value is calculated from a measured value and the detection limit/2 for the element. – data missing

Boatyards; *1* Hålkedalskilen, Strömstad,; *2* Nilssons båtbyggeri, Strömstad,; *3* Råssö, Strömstad; *4* Källviken, Uddevalla; *5* Dragsmark östra, Uddevalla; *6* Orust marina, Orust; *7* Rönnäng 1:75, Tjörn; *8* Rönnäng 1:153, Tjörn; *9* Toftenäs, Tjörn; *10* Vattenfall, Stenungsund; *11* Röd 1:73, Öckerö, *12* Fiskebäcks hamn, Göteborg; *13* Skintebo bryggor, Göteborg; *14* Bryggan 1, Halmstad; *15* Lomma hamn, Lomma; *16* Dragör, Malmö; *17* Skillinge hamn, Simrishamn; *18* Angelskog, Ronneby; *19* Hästö, Norrköping; *20* Lindö, Norrköping; *21* Strandängen, Nyköping; *22* Femöre 1:4, Oxelösund; *23* Bo Klok, Nynäshamn; *24* Tyresö strand 1:36, Tyresö; *25* Fisksätra marina, Nacka; *26* Kilsviken, Nacka; *27* Skutviken, Nacka; *28* Vikingarnas Segelsällskap, Stockholm; *29* Margretelund; Stockholm; *30* Årstaviken, Stockholm; *31* Fiskarfjärden, Skärholmen, Stockholm; *32* Göta Segelsällskap, Stockholm; *33* Björkdungens förskola, Danderyd; *34* Cirkusplatsen, Östhammar

**Table 5:** Organic compounds

Boatyard no	TBT Median (mg/kg DW)	TBT Max (mg/kg DW)	TPhT Median (mg/kg DW)	TPhT Max (mg/kg DW)	PAH carc. Median (mg/kg DW)	PAH carc. Max (mg/kg DW)	PAH ∑16 Median (mg/kg DW)	PAH ∑16 Max (mg/kg DW)	PCB 7 Median (mg/kg DW)	PCB 7 Max (mg/kg DW)
1	0.20	**40.00**	0.01	0.46	*1.30*	*2.40*	1.90	3.70	**–**	**–**
2	*1.30*	**42.00**	0.10	0.56	*0.97*	*1.50*	1.90	2.80	**–**	**–**
3	**–**	**–**	**–**	–	0.03	0.04	0.03	0.04	<0.025	**<0.025**
4	**–**	**–**	**–**	–	**10.04**	**21.44**	*21.20*	*41.00*	**17.30**	**27.00**
5	**–**	**–**	**–**	–	**–**	**–**	**–**	**–**	**–**	**–**
6	0.28	0.28	<0.001	<0.001	**–**	**–**	0.15	2.02	<0.008	<0.008
7	**2.50**	**17.10**	0.03	0.07	*2.00*	**180.00**	4.60	**340.00**	**0.61**	**1.90**
8	**–**	**–**	**–**	–	<1.1	<1.1	0.68	1.20	**–**	**–**
9	0.00	0.05	**–**	–	*0.36*	*4.00*	0.78	*8.50*	**–**	**–**
10	**–**	**–**	**–**	–	**–**	**–**	**–**	**–**	**–**	**–**
11	<0.001	<0.001	<0.001	<0.001	**–**	**–**	*40.70*	**223.60**	**–**	**–**
12	0.01	**2.50**			*0.60*	**15.40**	0.55	*23.00*	*0.03*	**1.60**
13	**40.10**	**51.00**	3.57	3.88	**–**	**–**	**–**	**´–**	**–**	**–**
14	**–**		**–**	–	**–**	**–**	5.40	**571.00**	**–**	**–**
15	0.32	**22.00**	0.03	1.70	**–**	**–**	**–**	**–**	**–**	**–**
16	*1.98*	**3.90**	**–**	–	**–**	**–**	*7.20*	**1,049.00**	**–**	**–**
17	0.14	**5.70**	0.00	0.02	**–**	**–**	0.26	*7.22*	**–**	**–**
18	**5.67**	**11.30**	0.02	0.04	**10.30**	**13.00**	*28.30*	*28.60*	**–**	**–**
19	**–**	**–**	**–**	–	*1.80*	**630.00**	4.25	**1480.00**	**–**	**–**
20	0.13	0.25	0.15	0.15	<0.002	<0.002	1.80	4.50	0.03	**0.28**
21	0.09	**4.00**	**–**	**–**	*1.40*	**79.00**	2.70	**170.00**	**–**	**–**
22	0.56	*1.60*	0.01	0.78	**–**	**–**	**–**	**–**	**–**	**–**
23	**–**	**–**	**–**	–	*0.56*	*3.70*	0.52	6.10	**–**	**–**
24	0.00	**0.21**	0.00	0.04	*1.40*	*1.40*	0.37	3.62	**–**	**–**
25	0.32	**0.64**	0.02	0.33	<0.30	*0.41*	0.30	0.86	0.00	**1.60**
26	**–**	**–**	**–**	**–**	*0.31*	**9.90**	0.84	*20.90*	**–**	**–**
27	**–**	**–**	**–**	**–**	*2.95*	**11.00**	**–**	**–**	**0.20**	**3.80**
28	**–**	**–**	**–**	**–**	*1.10*	*1.50*	3.10	4.10	**–**	**–**
29	**–**	**–**	**–**	**–**	*1.30*	**35.30**	3.80	**115.30**	**–**	**–**
30	**–**	**–**	**–**	**–**	*1.50*	*2.40*	4.40	6.70	**–**	**–**
31	**–**	**–**	**–**	**–**	*0.30*	*0.30*	1.00	1.30	**–**	**–**
32	**–**	**–**	**–**	**–**	*3.30*	**8.10**	*9.80*	*24.1*	**–**	**–**
33	**–**	**–**	**–**	**–**	**–**	**–**	**–**	**–**	**–**	**–**
34	**–**	**–**	**–**	**–**	**–**	**–**	1.88	**130.20**	<0.001	<0.001

The median and maximum concentrations of selected organic compounds in soil at 34 investigated boatyards in Swedish coastal municipalities. Values higher than the Swedish guideline values for sensitive land use (SL) are marked in italics and higher than less sensitive land use (LSL) are marked in bold. – data missing. The median value is calculated from a measured value and the detection limit/2 for the element. For the exact depth for each sample, we refer to the original reports

Boatyards; *1* Hålkedalskilen, Strömstad; *2* Nilssons båtbyggeri, Strömstad; *3* Råssö, Strömstad; *4* Källviken, Uddevalla; *5* Dragsmark östra, Uddevalla; *6* Orust marina, Orust; *7* Rönnäng 1:75, Tjörn; *8* Rönnäng 1:153, Tjörn; *9* Toftenäs, Tjörn; *10* Vattenfall, Stenungsund; *11* Röd 1:73, Öckerö, *12* Fiskebäcks hamn, Göteborg; *13* Skintebo bryggor, Göteborg; *14* Bryggan 1, Halmstad; *15* Lomma hamn, Lomma; *16* Dragör, Malmö; *17* Skillinge hamn, Simrishamn; *18* Angelskog, Ronneby; *19* Hästö, Norrköping; *20* Lindö, Norrköping; *21* Strandängen, Nyköping; *22* Femöre 1:4, Oxelösund; *23* Bo Klok, Nynäshamn; *24* Tyresö strand 1:36, Tyresö; *25* Fisksätra marina, Nacka; *26* Kilsviken, Nacka; *27* Skutviken, Nacka; *28* Vikingarnas Segelsällskap, Stockholm; *29* Margretelund; Stockholm; *30* Årstaviken, Stockholm; *31* Fiskarfjärden, Skärholmen, Stockholm; *32* Göta Segelsällskap, Stockholm; *33* Björkdungens förskola, Danderyd; *34* Cirkusplatsen, Östhammar

### Relation Between Guideline Values and Mean Concentrations

More detailed information of the mean values with standard deviation from each contaminant at each of the 34 boatyards is presented in Table 2 for the metals and Table 3 for organic substances. The variation in the data is very high, with coefficient of variations up to 378 % for Cu at boatyard 24 (Tables 2, 3).

The mean concentrations were very high in a range between 13 and 6,400 mg/kg DW for Cu, 18 and 1,746 for Zn, 4 and 5,396 for Pb, <0.001 and 39.92 for Hg, <0.05 and 2.3 for Cd, <0.001 and 40 for TBT, <.0.001 and 3.57 for TPhT, < 0.001 and 73.85 for carcinogenic PAHs, 0.03 and 157 for PAHs, and <0.001 and 17.32 for ∑7 PCB. The mean values of the means exceeded SL guidelines by, at the most for ∑7 PCB, a factor of 57. The corresponding figures for carcinogenic PAHs, Hg, Pb, TBT, Cu, ∑16 PAHs, Zn, and Cd were 31, 10, 9, 7, 6.7, 3.6, 1.6, and 1, respectively.

### Relation Between Guideline Values and Median Concentrations

The median values are presented in Table 4 for the metals and Table 5 for the organic compounds, and these values are frequently higher than the guideline values. The highest factor by which the mean median concentration among the contaminants studied exceeded SL was 400 for ∑7 PCB, with a mean value of 3 mg/kg DW. The corresponding factors for carcinogenic PAHs, TBT, Pb, Hg, Cu, ∑16 PAHs, Zn, and Cd were 6.8, 3.6, 2.9, 2.2, 1.7, 0.8, 0.7, and 0.7, respectively.

### Relation Between Guideline Values and Maximum Concentrations

The maximum concentration measured at each boatyard is presented in Table 4 for metals and Table 5 for organic substances. The concentrations are often very high, and the highest mean among the contaminants of the maximum concentrations is measured for ∑7 PCB of 6 mg/kg DW and thus exceeds SL 750 times. The corresponding factors for carcinogenic PAHs, Hg, Pb, Cu, ∑16 PAHs, TBT, Zn, and Cd were 155, 54, 53, 23, 23, 19, 6.8, and 3.4, respectively.

### Number and Percentages of Boatyards Where the Guideline Values Were Exceeded

Table 6 shows the number and percentage of boatyards where the guideline values were exceeded based on median, mean, and maximum values. The number of boatyards where the median concentration is higher than the guidelines for SL was lowest for Cd with 17 % (=5 sites) above SL and highest for carcinogenic PAHs, with 82 % (=18 sites) of the sites having higher concentrations (Table 6). The figures based on mean values show that the number of boatyards was lowest for Cd which was higher than SL at 33 % (=10 sites) and the highest value was for Pb, which was higher than SL at 82 % (=27 sites) of the boatyards (Table 6). Very high concentrations of contaminants were determined at most of the boatyards investigated. The maximum concentration was higher than SL at around 85 % of the boatyards for Cu, Pb, and carcinogenic PAHs. The lowest percentage was for ∑16PAHs, where SL was exceeded at slightly more than half of the boatyards (54 %).

**Table 6:** The number and percentage of boatyards where the median, mean, and maximum values were exceeding Swedish Guideline values of SL (Sensitive Land use) and LSL (Less Sensitive Land use)

	Cu	Zn	Pb	Hg	Cd	TBT	Canc PAH	∑16 PAH	∑7 PCB
No. of sampled boatyards	33	32	33	28	30	17	22	27	9
Guideline value for LSL (mg/kg DW)	**200**	**500**	**400**	**2.5**	**15**	**2**	**7**	**45**	**0.2**
Guideline value for SL (mg/kg DW)	*80*	*250*	*50*	*0.25*	*0.5*	*1*	*0.3*	*7*	*0.008*
Determinations based on median concentrations									
>LSL, no of boatyards	**7**	**3**	**4**	**1**	**0**	**3**	**2**	**0**	**3**
>SL, no of boatyards	*11*	*7*	*13*	*7*	*5*	*5*	*18*	*5*	*4*
% Where LSL is exceeded	21	9	12	4	0	18	9	0	33
% Where SL is exceeded	33	22	42	25	17	29	82	19	44
Determinations based on mean concentrations									
>LSL, no of boatyards	**16**	**7**	**9**	**6**	**0**	**7**	**6**	**4**	**5**
>SL, no of boatyards	*23*	*18*	*27*	*18*	*10*	*10*	*17*	*11*	*6*
% Where LSL is exceeded	48	22	27	21	0	41	27	15	56
% Where SL is exceeded	70	56	82	64	33	59	77	41	67
Determinations based on maximum concentrations									
>LSL, no of boatyards	**26**	**22**	**20**	**14**	**1**	**10**	**10**	**8**	**6**
>SL, no of boatyards	*28*	*25*	*28*	*20*	*20*	*11*	*19*	*15*	*6*
% Where LSL is exceeded	79	69	61	50	3	59	46	30	67
% Where SL is exceeded	85	78	85	71	67	65	86	56	67

The number of boatyards with measured values higher than the Swedish guideline for SL is marked in italics and higher than LSL is marked in bold

### Correlations

High correlations were found between the metals Cu, Zn, and Pb (Spearman′s pairwise non-parametric test: Cu and Zn, *R* = 0.74; Cu and Pb, *R* = 0.82; Zn and Pb, *R* = 0.73).

## Discussion

### Concentrations of Contaminants on Boatyards

The results clearly show that pleasure boatyards are often highly contaminated with high concentrations of several well-known toxic metals (Cu, Zn, Pb, Hg, and Cd) and organic compounds (i.e., TBT, TPhT, PCBs, and PAHs,) regardless of whether they are calculated as median, mean, or maximum values. Based on median values, the contaminants are higher than SL in 17–82 % of the boatyards; based on mean values, it is 33–82 % of the boatyards, where the contaminants exceed SL; and based on maximum values, 56–86 % of the boatyards have higher concentrations than SL (Table 6). This means that even if the concentrations vary between the different boatyards (Tables 1, 2, 3), it is clear that for a majority of sites the concentrations of many of the contaminants are higher than the guideline values for SL and often also higher than LSL and, therefore, these sites must be regarded as highly polluted areas. The risk increases in that not only one but also several well-known hazardous substances are found at the same place.

The most likely reason for the high variation among data is the unevenness of spread of paint flakes after boats are scraped and sand papered, in particular of the elements that derive from antifouling paints. This explanation of the high variation in boatyard soil has also been suggested by Turner (2013) in his study of the metals in two UK leisure boatyards and by Eklund et al. (2014) in an extensive study of one boatyard in Stockholm. Due to this unevenness in spread of paint residuals after scraping, the values of contaminants can vary greatly with a few extreme values enhancing the mean (compare data in Tables 2 and 3). For example, at boatyard 21, the mean value (*n* = 17) for Cu was 649 with a standard deviation of 1,490 but the median was only 25. This was caused by very high concentrations of Cu at three of the stations at boatyard 21, while much lower values were measured at the other 14 stations sampled at this site. Because of this, we consider the median value to be the more realistic way to present data from this dataset. In our study, the data are presented not only as means with standard deviation but also as median and maximum values from each boatyard to provide more complete information.

### The Origin of the Contaminants

The Cu, Pb, Zn, TBT, and TPhT most likely originated from the use of antifouling paints in which these compounds were used as active agents or booster compounds (e.g., Yebra et al. 2004, Thomas and Brooks 2010). Today, Cu is the most commonly used active agent in antifouling paints (Thomas and Brooks 2010). Zn is often used as a binder in antifouling paints, and the high correlation between Zn and the other two metals, Cu and Pb, (*R* > 0.73) further confirms the origin from antifouling paints. In the past, Hg was also extensively used in antifouling paints (Lunn 1974), which may explain the occurrence at least in deeper soil layers. PCBs were previously used in antifouling paints (Jensen and Renberg 1972) and may also derive from the use of plasticizer in the sealing of the deck and around windows, which may come off during maintenance work. It may also have originated from the leakage of oils containing PCBs from hydraulic systems. Since PCBs are highly persistent in the environment, Johnsen and Engöy (1999) concluded that the amount used several decades earlier could explain the high concentrations of PCB found in the sediment in a harbor in 1990s and this may be the case for PCB in the soil in boatyards.

Since the 1960s, TBT has been the main active compound in paint used in most countries (Fent 2006 and references therein). In some Asian countries, especially Japan, TPhT has also commonly been used as an active agent in antifouling paints (Horiguchi et al 1997, Fent 2006). In spite of prohibition in 1998 of organic tin compounds, TBT in particular is still found in very high concentrations in the surface soil at some boatyards. Our hypothesis is that older layers of paint that contained TBT come off during maintenance work and accumulate in the ground. This is supported by findings from the use of boat washers (similar to a car wash but where the boats are washed by rotating brushes while in the water) where very high TBT concentrations were measured in the collecting device beneath boat washers (Eklund et al. 2008, 2010). The same scenario may be true for PCBs and Hg, where trace concentrations may erode from boats with time. PAHs are produced mainly from the combustion of fossil fuels. High concentrations of PAHs found in the boatyards may result from washing boat motors and the localized automobile exhaust. Another potential PAH source is wood impregnated by creosote, which leaks PAHs.

### Regulations of the Contaminants

The high concentrations are surprising since several of these contaminants have been restricted or prohibited for many years or decades. Production of PCBs was already heavily restricted in the 1970s due to their carcinogenic effects. Today, PCBs are no longer used in most products (Directive 2004/850). Pb has been restricted since 1976 (Directive 76/769/EEC; Directive 89/677/EEC 1989; UN environment program 2008). In the beginning of the last century, the use of organo-mercury substances in antifouling paints was prohibited due to their negative health impacts (Yebra et al 2004). In 1979, Hg was restricted for use as biocide according to the plant protection Directive (79/117/EEG) and after that in many other special areas as e.g., use in cosmetics and toys.

Currently, there are no common European guidelines for safe TBT concentrations in soils or sediments. In water, concentrations of 0.0002 μg/l (Directive 2008/105/EC) are considered to be safe, and for the sediment, the proposed safe value is 0.02 μg/kg DW (Environmental Quality Standards (EQS) 2005). The high concentrations found in the boatyards in this and other studies (Decelis and Vella 2007) emphasize the need for EQS standards for these substances both in sediment and soil.

According to the EU Water Framework Directive (2000/60/EG), lead, mercury, cadmium, TBT, and PAHs are designated as priority hazardous substances that should be phased out as soon as possible but very high concentrations of these contaminants are still present in the soil at these boatyards. The risk is evident for leakage of these contaminants from the soil to the adjacent waters.

Today, hardly any regulations exist in any country with regard to the safety of boat maintenance (Turner 2010; Eklund et al. 2014). For example, in many countries, such as Sweden, no special permit is needed for boat clubs to maintain leisure boats on land. The data presented in this study further emphasize the need for more strict regulations. We propose that boat clubs should be required to announce their activities to environmental authorities. We also recommend that the management of boat clubs should be stricter and that rules are established on the maintenance of boats in order to minimize spread of contaminants.

### Risks to Humans from the Contaminants at Boatyards

Boat owners performing maintenance on their boat could potentially be exposed to the contaminants when either scraping boat hulls or damming in connection with sandpapering. The concentration of several elements and substances in the soil, regardless of whether the concentrations are based on median, mean, or maximum concentrations, is higher or much higher than what is considered to be acceptable for daily exposure (SL). For example, the measured maximum values exceed SL in 56–86 % of the boatyards, which means that the risk for exposure for one or more of the contaminants is high at most boatyards (Table 6).

Exposure to Pb may affect a number of processes in the heart, bones, reproductive system, and developing brain and nervous system (http://epa.gov/superfund/lead/health.htm). Nervous system exposure makes Pb particularly toxic to children, causing learning disorders. For Hg, the median value exceeded SL in 25 % of the boatyards and the maximum value was 71 %. Mercury may affect the brain, kidney, and lungs (http://www.epa.gov/hg/effects.htm). Cadmium can lead to damages in the kidney, bone, and lungs (Bernard 2008, http://www.occup-med.com/content/1/1/22).

Only limited data on the deposition of organo tin compounds in humans are available. Highest levels were found in humans with high consumption of seafood (Antizar-Ladislao 2008). In a study, blood samples from 38 volunteers were analyzed and concentrations up to 155 μg/l were detected (Kannan et al. 1999), which is comparable to levels in in vitro experiments with human blood cells causing immunotoxic effects (De Santiago and Aguilar-Santelises 1999). These findings show that people are exposed to TBT and the routes for exposure should be identified (Kannan et al. 1999). Our study indicates that one means of exposure may be through maintenance work on boat hulls with paint containing TBT.

Many of the PAHs are mutagenic (http://www.atsdr.cdc.gov/csem/csem.asp?csem=13&po=0), and PCBs are considered as probable human carcinogenic compounds (http://www.epa.gov/hudson/humanhealth.htm).

Based on the Swedish regulations, the maximum concentrations of the contaminants analyzed were 3–54 times greater for the metals (Cu, Zn, Pb, Hg, and Cd) and 19–155 times greater for organic substances (TBT, PCB, and PAHs) than concentrations that are not considered risks for the environment and the people who are in it every day [sensitive land use (SL)]. The maximum value of each element/substance from the entire dataset was much higher and the highest value of PCBs, for example, was 27 mg/kg DW and thus exceeded SL by a factor of 3,375, and the highest concentration of carcinogenic PAHs was 630 mg/kg DW, which exceeded the guideline SL concentration by a factor of 2,100. These high figures illustrate the potential risk for exposure at such places. Since the variation within the samples at each site is very high (a CV up to nearly 400 %) and the number of samples low, it is possible that even higher values may be found. Our findings are supported by similar studies that found high copper and zinc concentrations in spent paint particles from a marine leisure boat maintenance facility in the UK (Singh and Turner 2009a; Turner et al. 2009; Turner 2013; Eklund et al. 2014). These results make it likely that boatyards often are highly contaminated, a result that may be found around the world.

 Dust and fine eroded particles from antifouling paint can travel relatively long distances. Turner 2010 and the references therein and several reports show high levels of TBT contamination in nearby environments (Decelis and Vella 2007). In one case in Thailand, elevated lead levels were found in dust from a boatyard several hundred meters from the boatyard (Maharachpong et al. 2006).

The direct and indirect health and environmental risks are difficult to estimate. Since the measured concentrations for all analyzed elements and organic compounds exceeded the acceptable concentrations by several factors, boatyards should be considered dangerous. The combination of many well-known harmful substances enhances the risk for human exposure, and we recommend that measures be taken to minimize human risks and prevent the spread of the harmful substances from boat maintenance facility areas. The high risk is evident for harmful exposure at boatyards, and children, in particular, should not be at such sites.

### Effects on the Environment

Data regarding the effects of contaminated boatyard soils on organisms have only been found in one recent study by Eklund et al. (2014). In this study, soil from a boatyard, which contained contaminants in high concentrations similar to those in the compilation, was used for producing leachate water. These waters were tested on three organisms: the bacterium *Vibrio fischeri*, the macroalga *Ceramium tenuicorne,* and the crustacean *Nitocra spinipes*. The leachates were shown to be toxic to all the three test organisms. Several other studies have reported high concentrations of the same substances in harbor sediments (for example, Eklund et al. 2008, 2010) and negative effects from harbor sediment to test organisms (Eklund et al. 2010), and sediment paint particle exposure on algae and benthic invertebrates (Turner et al. 2008a, b, 2009a, c). The occurrence of organotin from antifouling paints and the effects in the aquatic environment have been reviewed by Hoch (2001), Fent (2006) and Antizar-Ladislao (2008). These data may be relevant since it is likely that many of the accumulated contaminants from the soil will eventually end up in harbor sediments. The conclusion is that leakage from boatyards likely contains many pollutants toxic to species living in water.

### Possible Spread of Contaminants

Although most of the substances found at high concentrations in boatyards have been restricted or forbidden for a long time, the data show that these substances are still found in the soil and in the environment (e.g., Decelis and Vella 2007; Strand et al 2003; Eklund et al 2008, 2010; Turner 2010 and references therein).

There are two possible processes in which toxic metals are released from soils. The first process occurs when the metals are washed out by rain water into the adjacent waters. The second process occurs when the metals penetrate deeper into the ground. For organic substances, degradation may be another possible explanation. Most likely, a combination of these processes occurs.

To our knowledge, soil contaminant leaching and mobility studies have not been conducted around boatyards. However, this knowledge is essential for estimating the risk that these contaminated boatyards may pose for the aquatic environment. Some information may be obtained from copper and zinc leakage studies from paint particles (Singh and Turner 2009b) and paints applied to boat hulls (Ytreberg et al. 2010).

The distribution and leakage of elements and organic substances depend to a great extent on the characteristics of the soil, such as pH, organic matter content, mineralogical composition, redox potential, and the presence of microorganisms (de Carvalho Oliveira and Santelli 2010). While this is beyond the scope of this study, it should be further studied and the composition and effects of compounds from boatyards should be further investigated.

### The Extent of the Problem of Contaminated Boatyards

The data compiled in this study are from the southern part of Sweden where most pleasure boats have their home harbor (Swedish Transport Agency 2010). It contains all existing investigations from 66 of the total 82 coastal municipalities in Sweden. The result is thus considered to be representative for boatyards in Sweden. Comparable regulations apply, in particular, for organic tin compounds in other European countries as well as in North America, Australia, and New Zealand (e.g., Antizar-Ladislao 2008; Dafforn et al 2011 and references therein), and the results might, therefore, be applicable to boatyards in these countries.

The problem of contaminated boatyards has so far been neglected by the environmental authorities, and investigations have only been performed in one-third of the coastal municipalities in southern Sweden. The rest of the municipalities did not regard boatyards prioritized objects to investigate. Considering that most of the ca 1 million boats in Sweden are found in southern part (Swedish Transport Agency 2010), a rough estimation on around 500,000 boats placed at boatyards with 200 boats at each gives 2,500 such boatyards. With an extrapolation based on the 6 million leisure boats in Europe (ICOMIA 2007), there should be approximately 6,000 boatyards.

The reason for performing these 34 investigations was most often (18 sites) due to a planned change in land use (for example, building residential housing instead of using the area as a boatyard). Thirteen of these had been risk-assessed, and the remaining boatyards are in the evaluation process. For all of the assessed boatyards, the municipalities have decided on remediation of the soil. Since the reason for the decision is a change of use of the land from boatyard to building residential houses, the criteria have been that the concentrations should be below the recommended values for SL. In all cases, this means removal of all contaminated soil. Since boatyards often are situated at attractive living places near shore sites, this will most likely occur in several boatyards in the future, not only in Sweden but also in many other countries.

## Conclusions

Despite strict regulations, boatyard soils are still heavily contaminated with a number of well-known dangerous metal and organic substances, such as Cu, Zn, Pb, Hg, Cd, TBT, TPhT, PAHs, and PCBs. Of these, Hg, Pb, Cd, TBT, and PAHs are all classified as hazardous priority substances, which according to the European Water Frame Directive should be phased out. In general, the same pattern of contaminants was found in all the 34 boatyards investigated. Since the regulations for using antifouling paints have been similar in the EU, these findings are probably valid for boatyards around Europe. Comparable regulations for organotin compounds exist in Europe, North America, Australia, and New Zealand, and the situation in their boatyards might be similar for these compounds.

We recommend stricter regulations for boat maintenance to reduce the addition of and to minimize any further spread of toxic substances in connection with maintenance in boatyards. Today, no special permit is needed for boat clubs to designate a maintenance facility area. We suggest that these boat clubs should be investigated to determine if they should be obliged to obtain approval by environmental authorities to run a boatyard. We finally argue that non-toxic methods should be used to prevent fouling to reduce the spread of toxic substances from leisure boats.

## Electronic supplementary material

Below is the link to the electronic supplementary material.

Supplementary material 1 (DOCX 19 kb)
