# Supplementary material for: Pleasure Boatyard Soils are Often Highly Contaminated
Source: Environ Manage. 2014 Feb 23;53(5):930–46. doi: 10.1007/s00267-014-0249-3 (PMC3972443; doi:10.1007/s00267-014-0249-3)
Supplement: Supplementary file 1 — Supplementary material 1 (DOCX 19 kb) [file 267_2014_249_MOESM1_ESM.docx]

Supplementary material

*List of municipalities where investigations have been performed on boat yards and the respective web sites where the reports can be obtained.*

| Municipality and their web site | Boatyard and year of investigation | Year of investigation |
| --- | --- | --- |
| Strömstad  [kommun@stromstad.se](mailto:kommun@stromstad.se) | Hålkedalskilen | 2008 |
|  | Nilssons båtbyggeri, | 2007, 2009 |
|  | Råssö | 2003 |
| Uddevalla  [kommunen@uddevalla.se](mailto:kommunen@uddevalla.se) | Källviken | 2003 |
|  | Dragsmark east | 2003 |
| Orust  [orustkommun@orust.se](mailto:orustkommun@orust.se) | Orust marina | 2010 |
| Tjörn  [kommun@tjorn.se](mailto:kommun@tjorn.se) | Rönnäng 1:75, | 2008 |
|  | Rönnäng 1:153 | 2010 |
|  | Toftenäs 1:15; 1:31 | 2008 |
| Stenungsund,  [kommun@stenungsund.se](mailto:kommun@stenungsund.se) | Vattenfall | 2003 |
| Öckerö,  [kommun@ockero.se](mailto:kommun@ockero.se) | Röd 1:73 | 2010 |
| Göteborg,  [stadsledningskontoret@stadshuset.goteborg.se](mailto:stadsledningskontoret@stadshuset.goteborg.se) | Fiskebäcks hamn | 2010 |
|  | Skintebo bryggor | 2009 |
| Halmstad,  [halmstad.kommun@halmstad.se](mailto:halmstad.kommun@halmstad.se) | Bryggan 1 | 2011 |
| Lomma,  [kommunstyrelsen@lomma.se](mailto:kommunstyrelsen@lomma.se) | Lomma hamn | 2010 |
| Malmö,  [kommunstyrelsen@malmo.se](mailto:kommunstyrelsen@malmo.se) | Dragör | 2009 |
| Simrishamn, [kommunledningskontoret@simrishamn.se](mailto:kommunledningskontoret@simrishamn.se) | Skillinge hamn | 2007 |
| Ronneby,  [stadshuset@ronneby.se](mailto:stadshuset@ronneby.se) | Angelskog | 2006 |
| Norrköping,  [norrkoping.kommun@norrkoping.se](mailto:norrkoping.kommun@norrkoping.se) | Hästö | 2005 |
|  | Lindö | 2009 |
| Nyköping,  [kommun@nykoping.se](mailto:kommun@nykoping.se) | Strandängen | 2002, 2004 and 2006 |
| Oxelösund,  [kommun@oxelosund.se](mailto:kommun@oxelosund.se) | Femöre 1:4 | 2007 |
| Nynäshamn,  [kommunstyrelsen@nynashamn.se](mailto:kommunstyrelsen@nynashamn.se) | Bo Klok | 2003 |
| Tyresö  [kommun@tyreso.se](mailto:kommun@tyreso.se) | Tyresö strand 1:36 (1:35) | 2004 |
| Nacka,  [info@nacka.se](mailto:info@nacka.se) | Fisksätra marina | 2011 |
|  | Kilsviken | 2006 |
|  | Skutviken | 2008 |
| Stockholm stad,  [kommunstyrelsen@stockholm.se](mailto:kommunstyrelsen@stockholm.se) | Vikingarnas Segelsällskap (Djurgården) | 1997 |
|  | Margretelund  (Ulvsundasjön) | 1997 |
|  | Årstaviken | 1997 |
|  | Fiskarfjärden (Skärholmen) | 1997 |
|  | Göta Segelsällskap (Långholmen) | 1997 |
| Danderyd,  [kommunen@danderyd.se](mailto:kommunen@danderyd.se) | Björkdungens förskola | 2006 |
| Östhammar,  [kommunen@osthammar.se](mailto:kommunen@osthammar.se) | Cirkusplatsen | 2008 |
